# Lornoxicam-Loaded Chitosan-Decorated Nanoemulsion: Preparation and In Vitro Evaluation for Enhanced Transdermal Delivery

**DOI:** 10.3390/polym14091922

**Published:** 2022-05-09

**Authors:** Rahman Ullah Khan, Shefaat Ullah Shah, Sheikh Abdur Rashid, Faiza Naseem, Kifayat Ullah Shah, Arshad Farid, Khalid Rehman Hakeem, Majid Rasool Kamli, Eman Hillal Althubaiti, Soha A. Alamoudi

**Affiliations:** 1 Skin/Regenerative Medicine and Drug Delivery Research, GCPS, Faculty of Pharmacy, Gomal University, Dera Ismail Khan 29050, Pakistan; rahmanhamid444@gmail.com (R.U.K.); shefaatbu@gmail.com (S.U.S.); faizanaseem17@gmail.com (F.N.); kifayatrph@gmail.com (K.U.S.); 2 Gomal Center of Biochemistry and Biotechnology, Gomal University, Dera Ismail Khan 29050, Pakistan; arshadfarid@gu.edu.pk; 3 Department of Biological Sciences, Faculty of Science, King Abdulaziz University, Jeddah 21589, Saudi Arabia; kur.hakeem@gmail.com (K.R.H.); hakeem.bot@gmail.com (M.R.K.); 4 Princess Dr. Najla Bint Saud Al-Saud Center for Excellence Research in Biotechnology, King Abdulaziz University, Jeddah 21589, Saudi Arabia; 5 Department of Public Health, Daffodil International University, Dhaka 1341, Bangladesh; 6 Center of Excellence in Bionanoscience Research, King Abdulaziz University, Jeddah 21589, Saudi Arabia; 7 Department of Biotechnology, College of Science, Taif University, P.O. Box 11099, Taif 21944, Saudi Arabia; i.althubaiti@tu.edu.sa; 8 Department of Biological Sciences, Science and Arts College, King Abdulaziz University, Rabigh 25726, Saudi Arabia; saalamudi@kau.edu.sa

**Keywords:** lornoxicam, nanoemulsion formulation, chitosan, in vitro drug release, ex vivo permeation, thermodynamic stability

## Abstract

Nanoemulsions are promising drug delivery systems for the administration of poorly soluble drugs like lornoxicam (LRX) by oral or parenteral routes. Such formulations work perfectly for transdermal delivery of lornoxicam-type drugs. It has also been established that formulating such a delivery system is highly dependent on the presence, type, and concentration of excipients taking part in the formulation. The inherent characteristics of nanoemulsion (NE), i.e., smaller globule size and excipient nature, facilitate the drug’s passage through skin. The current study was aimed at the development of an NE-based formulation of LRX to improve the drug solubility in vitro as well as to enhance drug skin permeation to promote therapeutic outcome in appropriate time. Spontaneous self-emulsification technique was utilized to develop optimized LRX-encapsulated NE-based formulations. ATR-FTIR spectra of the pure drug and various formulations did not show any interaction between the drug and various formulation excipients showing compatibility. Globule size for stable formulations ranged between 63–168 nm. These formulations were characterized for viscosity, surface tension, pH, drug encapsulation efficiency, in vitro drug release, and drug skin permeation studies. Chitosan-decorated optimized NE formulation of LRX showed about 58.82% cumulative drug release, showing an anomalous non-Fickian diffusion mechanism of drug release. Drug encapsulation efficiency, in vitro drug release, and skin permeation studies exhibited promising results. An appreciable drug entrapment efficiency was exhibited by optimized NE formulations LRX-6, 71.91 ± 3.17% and C-LRX, 65.25 ± 4.89%. Permeability parameters like enhancement ratio (Er), permeability constant (Kp), and steady state flux (Jss) showed higher values and exhibited good results based on formulation type. The selected promising formulation type “LRX-6” showed significantly different results as compared to other formulations (LRX-4, 5, and 7). The skin permeation property of the LRX-6 formulation was compared to similar chitosan-based formulations and was found to have better skin permeation results than chitosan-based formulations. This study clearly exhibited that an LRX-containing NE-based formulation can be formulated to form a stable drug delivery system. Such formulations are promising in terms of physicochemical characteristics, improved solubility, and high skin permeation potential.

## 1. Introduction

The use of nonsteroidal anti-inflammatory drugs (NSAIDs) for the treatment of inflammatory illnesses such as rheumatoid arthritis and osteoarthritis is widely advised. NSAIDS are effective in treating inflammatory conditions when taken orally. However, NSAIDs’ therapeutic usage has been limited due to side effects such as gastrointestinal mucosa irritation and ulceration [1]. Transdermal administration of NSAIDS is one method of avoiding toxicity and allowing them to be used for extended periods of time [2]. By delivering medications into systemic circulation through the skin, transdermal methods avoid changes in absorption rate, metabolism, and gastrointestinal side effects that can occur with oral drug delivery [3]. This is effective for treating chronic illnesses [4]. This enables the delivery of potent drugs with the added benefits of self-administration and improved therapeutic effectiveness [5]. Lornoxicam (LRX) is an oxicam-class NSAID with a high potency. Its chemical formula is C_13_H_10_ClN_3_O_4_S_2_. It is widely used in the treatment of chronic pain and inflammation. Oral use of LRX, like other NSAIDS, causes a slew of renal, gastrointestinal, and hematological side effects [6]. Aside from that, due to its short half-life of 3–4 h, it requires regular administration. Furthermore, parenteral administration is not advised in the case of chronic illnesses [7]. Controlled drug delivery of LRX via the transdermal route is a good way to avoid all of the problems that occur with LRX administration. Naturally, chitosan is derived by deacetylation of chitin. This polymer is inert, biodegradable, and biocompatible, so it is widely employed in a variety of pharmaceutical formulations [8]. LRX has been developed as a chitosan-decorated nanoemulsion formulation in this work. The skin serves as a good barrier against transdermal drug penetration. To improve transdermal drug delivery, many chemical enhancers have been devised. These chemical enhancers promote drug molecule flux by interacting with skin components [9]. These drugs modify the skin’s barrier function in a reversible way, allowing poorly penetrating molecules to penetrate through the stratum corneum and into systemic circulation [10]. Various studies have shown that when penetration enhancers are used together, they can have a synergistic effect, enhancing skin permeation more effectively than when they are used alone [11]. In transdermal formulations, co-solvents have been widely employed as carriers and penetration enhancers. These chemicals not only boost drug solubility, but they also change the structure of the skin, increasing penetration rate. As a result, both permeability and drug release are affected [12]. Apart from being utilized as an oil phase, almond oil has been used to increase percutaneous drug absorption. In several transdermal formulations, almond oil and polyethylene glycol (PEG 400) proved to be a highly beneficial combination [13]. Therefore, the current study’s goal is to improve LRX’s transdermal permeability by employing PEG 400 as a co-solvent and almond oil as a penetration enhancer and oil phase. The chitosan adorned nanoemulsion formulation of LRX (C-LRX NE) has been formulated for this purpose using a spontaneous emulsification process that can regulate a constant drug release rate for a duration of 24 h.

## 2. Materials and Methods

### 2.1. Materials

LRX was kindly gifted by Hilton Pharma Pvt. Ltd., Pakistan. Other chemicals/excipients used were low molecular weight chitosan (degree of deacetylation 75–85%, 50–190 kDa), Tween 80, PEG 400 (monofunctional) (Sigma-Aldrich Co., 3050 Spruce Street, St. Louis, MO 63103, USA, +13-14-771-5765), almond oil (Marhaba Laboratories, Punjab, Pakistan), and triethanolamine (BDH, Poole, UK). All the chemicals used in this study were of analytical grade and were used without further purification.

### 2.2. Pre-Formulation Studies

#### 2.2.1. Solubility Studies

Solubility studies are considered a basic standard for screening oils, surfactants, and co-surfactants for the development of nanoemulsions, which present high dissolution and excellent rate and degree of skin permeation. To investigate the solubility of LRX, a saturated solubility experiment was performed by mixing an excess quantity of drug with the specified amount of selected oils, surfactants, and co-surfactants. The mixture was continuously stirred for 72 h at 25 °C to ensure complete dissolution. At the end, the vial contents were centrifuged at 5000 rpm for 15 min to separate insoluble drug segments. The solution was filtered using 0.45 µm filter paper to remove any insoluble residues. The clear solution was examined under UV spectrophotometer (UV-2600i, Shimadzu, Japan) at 376 nm wavelength [14]. All experiments were performed in triplicate.

#### 2.2.2. Drug Excipient Compatibility Study (ATR-FTIR Analysis)

Pure LRX, chitosan powder, PEG 400, Tween 80, and NE formulation with and without chitosan were subjected to ATR-FTIR spectroscopy (Perkin Elmer, Waltham, MA, USA). This study was performed in order to investigate the compatibility profile of the polymer with the formulation components as well as to determine the wavenumbers and functional groups present in the formulations. Diamond crystal was employed for the placement of ingredients as well as formulation samples and then subjected to pressing by means of knob. The spectrum of each sample was recorded in the 400–4000 cm^−1^ wavenumber range. Triplicate readings were taken [15].

### 2.3. Preparation of LRX-NE Formulations with and without Chitosan

NE formulation and its optimization was achieved with a spontaneous emulsification technique [15,16]. Briefly, o/w NE formulation of LRX was constituted by dropwise addition of oil phase into the aqueous phase. To generate the oil phase, adequate proportions (*w*/*w* %) of almond oil and LRX were homogenized at 2000 rpm for at least 60 min, followed by dropwise addition of PEG 400 as co-surfactant and further homogenized for an hour to diminish surface strain. Aqueous phase was prepared by mixing deionized water and Tween 80 (non-ionic surfactant, HLB = 15) and blended (2000 rpm) for two hours. For the preparation of NE, both phases were mixed by adding oil phase dropwise into the aqueous phase and agitated (2000 rpm) for 10 min. Later, the mixture was homogenized at high speed (4000 rpm) for two minutes to produce the fine NE. All procedures were carried out at 25 °C [17].

Similarly, chitosan-decorated NEs were prepared by adding the requisite amount of chitosan (low molecular weight, ~50 kDa) to water along with two drops of glacial acetic acid to optimize the viscosity of the polymer solution. The mixture was homogenized at 2000 rpm for 24 h to ensure complete homogenization. After, Tween 80 was added and again blended for 20 min. Then, the oil phase (LRX mixed with almond oil as explained above) was added dropwise to the aqueous phase and agitated (2000 rpm) for 10 min. The resultant mixture was homogenized at high speed (4000 rpm) for two minutes to produce the fine NE. High speed homogenization was performed to achieve formulation homogeneity.

### 2.4. Physico-Chemical Characterization of LRX-NE and C-LRX NE Formulations

#### 2.4.1. Thermodynamic Stability Assessment of LRX-NE Formulations with and without Chitosan

The optimized formulations of both LRX-NE as well as C-LRX NE were investigated under stress conditions for 28 days as per ICH guidelines to analyze their thermodynamic stability profiles. The protocol of this study was followed as per the previously reported study with some modifications as follows [18]:

- Heating–cooling cycle

The optimized NE formulations containing LRX alone as well as chitosan were initially placed in the incubator at 40 °C for 28 days, followed by their cooling to room temperature. This test was performed to observe the formulation’s physical appearance as well as any evidence of creaming or turbidity.

b. Centrifugation

This test involved subjecting optimized formulations with and without chitosan to centrifugation (D3024, SCILOGEX, Rocky Hill, CT, USA) at a speed of 5000 rpm for 10 min and checking for any visible signs of phase separation.

c. Freeze–thaw cycle

The optimized LRX formulations with and without chitosan were assessed by freeze–thaw cycle method for 28 days by placing them in a deep freezer (2 °C), followed by holding them at room temperature. This extreme treatment was performed to note whether the formulations would return to their original form or not.

#### 2.4.2. pH Measurements

The pH levels of LRX-NE formulations with and without chitosan were measured using a pH meter (UB-10, DENVER, Germany) after calibration using standard buffer solutions at 25 °C. Measurements were taken in triplicate.

#### 2.4.3. Globule Size Measurement

Globule size and size distribution of LRX-NE formulations with and without chitosan were determined by Zetasizer (Zetasizer Pro, Malvern, UK). This system utilizes the guideline of variances in light dispersing due to “Brownian movement” of the particles. About 2 mL of NE formulation was meticulously diluted with 50 mL of deionized water and an aliquot (2 mL) of this dilution was subjected to Zetasizer analysis [19]. Light dissipation was observed at a temperature of 25 °C at an angle of 90°.

#### 2.4.4. Zeta Potential Analysis

The surface charge of NE formulations was calculated utilizing a Zetasizer (Zetasizer Pro, Malvern, UK) in zeta potential cell. Approximately 700 µL of formulation was incorporated in the cell cuvette of the Zetasizer. The estimation of zeta potential typically increases from −30 mV to +30 mV. The nanoemulsion formulations demonstrating their zeta potential values within ±30 mV are considered thermodynamically stable with homogenous distribution of droplet size. NE samples were placed in clear expendable zeta cells and results were noted. All the cuvettes were washed with methanol and flushed before each test.

#### 2.4.5. Drug Content and Drug Entrapment Efficiency

Drug content and drug entrapment efficiency were determined with an indirect method using UV spectroscopy. The dilution of the NE was performed suitably (1:10) with ethanol to achieve the desired drug concentration of 10 μg/mL. The absorbance was noted by UV spectrophotometer (UV-2600i, Shimadzu, Japan) at 376 nm [14]. All experiments were performed in triplicate. Drug content was determined by the following Equation (1) [20]:

(1) Drug Content = Drug in supernatant + Drug in sediment.

Similarly, the following Equation (2) was used to calculate drug entrapment efficiency [21]:

(2) Drug entrapment efficiency =[(added drug − free drug / added drug)]×100

#### 2.4.6. Viscosity and Surface Tension Measurement

The viscosity measurement of undecorated LRX-NE as well as C-LRX NE formulations was carried out with a Brookfield viscometer (Ametek Brookfield dv2t, Ametek Brookfield, Middleborough, MA, USA) at room temperature [16]. A specified volume of undiluted sample was placed for measurement using spindle no. 63 at various rotational speeds (rpm) at room temperature (25 ± 0.5 °C). Similarly, surface tension was determined by using a stalagmometer. All investigations were performed in triplicate.

#### 2.4.7. Surface Morphology

The morphological attributes of LRX-NE formulations with and without chitosan were investigated by employing transmission electron microscopy (TEM; Hitachi H-6000, Hitachi, Tokyo, Japan). In this case, a specific quantity of each formulation (3 µL) was kept on a grid coated with carbon and allowed to stand for time period of 5 min. The carbon-coated grid was then subjected to blotting by means of filter paper and subsequent staining with phosphotungstic acid (2%). This was followed by observing the sample at a voltage of 100 kV and taking images at various resolutions [22].

#### 2.4.8. In Vitro Drug Release and Kinetic Model Profiling

An in vitro drug release experiment was conducted as per the previously reported study [18]. For this purpose, a Franz diffusion cell was employed, which is composed of 2 compartments: donor and receptor with 3 mL and 6 mL capacity, respectively. Before the placement of samples containing LRX-NE formulations with and without chitosan decoration, the temperature and stirring speed were kept at 32 ± 0.5 °C and 300 rpm, respectively. Artificial membranes were employed for the analysis of in vitro drug release so, for this purpose, a Tuffryn membrane (Sartorious, Göttingen, Germany) was fixed between the donor and receptor compartments. The donor compartment was loaded with formulation sample (1 g), while the receptor compartment was filled with sodium acetate buffer (pH 5.5). A sample volume of 2 mL was withdrawn from the receptor compartment by means of spiral syringe after specified time periods of 0, 1, 2, 4, 8, 12, 18, and 24 h and was then replaced by fresh buffer in order to maintain sink conditions in the receptor compartment. The collected samples were subjected to UV spectrophotometer analysis at 376 nm, and hence drug release behavior was investigated. In order to investigate the drug release pattern, the data of drug release was input into the Korsmeyer-Peppas model [7].

#### 2.4.9. Permeation Studies

In this study, a rabbit was employed as a test animal and preparation of its skin was performed in order to determine ex vivo permeation behavior. A male rabbit of the desired weight (1–1.5 kg) was purchased from a local market in Dera Ismail Khan district, Pakistan. Hairs from the rabbit’s dorsal region were removed by means of hair-removing cream. The rabbit was then subjected to sacrifice by means of cervical dislocation. Excision of dorsal skin was performed with a surgical blade. The skin was then thoroughly washed and defatted. The defatted skin was then wrapped in aluminum foil and kept in a refrigerator for later use. On the day of experiment, its removal from the refrigerator and subsequent soaking in lukewarm water for 1 h was performed. Then, an ex vivo permeation experiment was carried out by clamping the skin in a Franz diffusion cell. Approval of this study, reference number (851/QEC/GU), was assigned by the Ethical Review Board of Gomal University, Dera Ismail Khan, Pakistan. The ex vivo drug permeation experiment was carried out as per the previously reported study [18]. For this purpose, a Franz diffusion cell was employed, which is composed of 2 compartments: donor and receptor with 3 mL and 6 mL capacity, respectively. Before the placement of samples containing LRX-NE formulations with and without chitosan decoration, the temperature and stirring speed were kept at 37 ± 1 °C and 300 rpm, respectively. Animal skin is generally employed for the analysis of ex vivo permeation, so, for this purpose, rabbit skin (previously prepared) was fixed between the donor and receptor compartments. The donor compartment was loaded with formulation sample (1 g), while the receptor compartment was filled with phosphate buffer solution (pH 7.4). A sample volume of 2 mL was withdrawn from the receptor compartment by means of spiral syringe after specified time periods of 0, 1, 2, 4, 8, 12, 18, and 24 h, and was then replaced by fresh buffer in order to maintain sink conditions in the receptor compartment. The collected samples were subjected to UV spectrophotometer analysis at 376 nm and hence drug release behavior was investigated [23].

#### 2.4.10. Permeation Data Analysis

The permeation analysis was performed by plotting the total quantity of LRX that was absorbed/unit membrane area (µg cm^−2^) against time [16]. Linear regression analysis was performed in order to calculate the steady state flux (J_ss_, µg cm^−2^ hr^−1^) of LRX by using the slope of the plot. The permeability co-efficient (Kp) of the drug through the stratum corneum was determined from the following Equation (3):

(3) $$K_{p}={\;J}_{\mathrm{ss}}\;/\;C$$

where C = initial concentration of the drug in the donor compartment.

The penetration enhancing effect was calculated in terms of enhancement ratio (ER) as follows [24]:

(4) $$\mathrm{ER}\;={\;J}_{\mathrm{ss}\;}\mathrm{of}\;\mathrm{formulation}\;/{\;J}_{\mathrm{ss}}\;\mathrm{of}\;\mathrm{control}$$

### 2.5. Statistical Analysis

Data has been presented as mean ± SD followed by one-way analysis of variance (ANOVA). Following ANOVA, an appropriate post hoc Tukey’s test was also performed. Dunnett’s *t*-test (*p* < 0.001) was considered significant.

## 3. Results

### 3.1. Solubility Study

LRX solubility in different oils and surfactants as well as co-surfactants was investigated by dissolving an excess of the drug in a specified volume of carefully shortlisted oils, surfactants, and co-surfactants as shown in Table 1. Almond oil was chosen as oil phase for formulating NEs. Reasons include appropriate drug solubility and effect on skin permeation [24,25]. The chief constituent of almond oil is monounsaturated oleic acid that not only served as an oil phase, but also exhibited pronounced skin permeation enhancer potential. This synergistic effect facilitates the formulation’s preparation and functioning.

Although Cremophor RH 40 showed higher solubility for LRX than Tween 80, Tween 80 was chosen as the surfactant due to being non-ionic in nature, offering less toxicity and more miscibility with the formulation ingredients. An appreciable drug solubility profile and pivotal role in skin permeation are also reported in the literature [24,25,26].

Drug solubility in tested co-surfactant molecules was found better in DMSO and PBS (Table 1), but miscibility of these molecules with NE composition without interfering with stability was not possible. Consequently, PEG 400, a short-chain alcohol, was chosen as co-surfactant molecule. It has been used as a co-surfactant in emulsion formulations, having the additional benefit of co-solvent action.

### 3.2. ATR-FTIR Analysis

**Figure 1:**
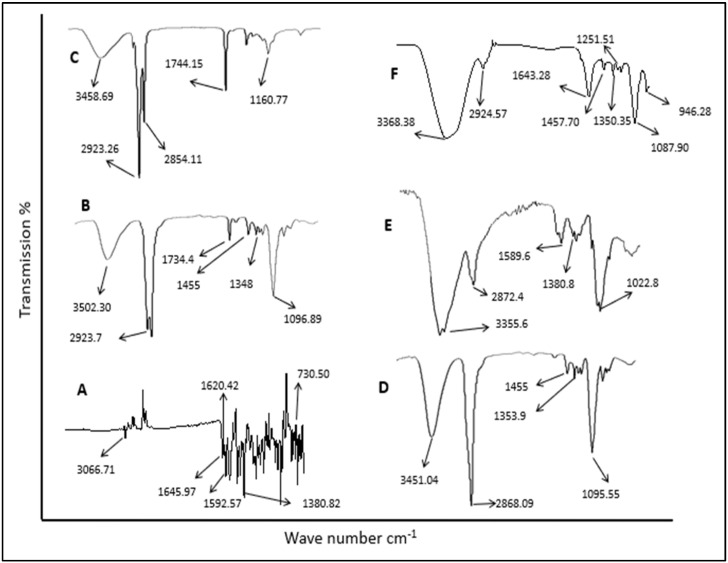
ATR-FTIR spectra of (**A**) pure drug, (**B**) Tween 80, (**C**) almond oil, (**D**) PEG 400 (**E**) chitosan, and (**F**) chitosan-decorated LRX-NE.

The possibility of interactions that occur between LRX (pure drug) and formulation excipients (chitosan, Tween 80, PEG 400 and almond oil) in NE formulations was investigated by means of ATR-FTIR spectrometer (Spectrum 100, Perkin Elmer) using a MIRacle ATR accessory (PIKE Technologies, Madison, WI, USA). The ATR-FTIR spectra of LRX, formulation excipients, and chitosan-decorated LRX-NE formulations are presented in Figure 1. The ATR-FTIR spectrum of LRX showed characteristic peaks at 1645 cm^−1^; 1620 cm^−1^ and 1592 cm^−1^; 1143–1380 cm^−1^; and 730 cm^−1^ which indicate primary amide stretching vibration, N-H group bending vibration, O=S=O stretching vibration, and C-Cl stretching vibration, respectively [27]. A characteristic band peak at 3066 cm^−1^ was also observed, which indicated C-H aromatic ring stretching vibration. Likewise, the ATR-FTIR spectra of chitosan exhibited bands at 3355 cm^−1^, 2872 cm^−1^, and 1022 cm^−1^, which indicated O-H and NH_2_ stretching vibration, C-H aliphatic stretching, and C-O-C glycosidic linkage, respectively. C-LRX NE formulation exhibited band shifting, attenuation or disappearance of pure LRX, showing strong physical contact of the drug with polymer, hence confirming good compatibility between drug and polymer. There was an absence of any new band formation in all formulations, suggesting no chemical interactions between drug and polymer [28].

### 3.3. Preparation of LRX-NE Formulations with and without Chitosan

Different NE formulations of LRX were prepared with varying concentrations of oil and surfactant molecules (Table 2). In total, nine different formulations were prepared by maintaining a surfactant to co-surfactant ratio of 2:1. Samples were prepared with a constant premix (oil and surfactant mixture) ratio with aqueous phase (1:10) and a fixed quantity of LRX having been added in all formulations. LRX-1, 2, and 9 exhibited phase separation while standing, while LRX-3 and 8 remained turbid and did not form transparent formulations. Therefore, these formulations were not included in further investigations. LRX-4, 5, 6, and 7 displayed transparent appearances and generally remained stable during the standing period. The reason could be a good blend or ratio of oil and surfactant molecules in these formulations as compared to other formulations that had either higher ratios of oil phase or surfactant molecules. Owing to its skin-friendly and stable characteristics, the LRX-6 formulation was designated as our optimized formulation. A C-LRX NE formulation was fabricated in comparison to the optimized LRX formulation (LRX-6) to investigate the impact of chitosan on skin permeation.

### 3.4. Physicochemical Characterization of LRX-NE with and without Chitosan

#### 3.4.1. Thermodynamic Stability

NEs should exhibit stability at different temperatures and should have the ability to preserve spontaneous emulsification upon dilution. Therefore, thermodynamic stability experiments were undertaken. Optimized LRX-NE formulations and C-LRX NE were subjected to different thermodynamically stressed conditions. Any variations in odor, color, or appearance were examined and were then compared to the blank NE formulation. The NE formulation passed a centrifugation test. Creaming, turbidity, and phase separation were absent (Table 3).

#### 3.4.2. Surface Morphology Analysis

**Figure 2:**
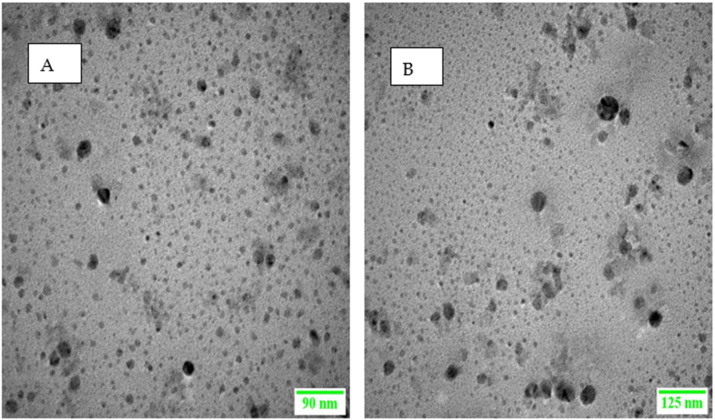
TEM images of (**A**) LRX-loaded NE (**B**) C-LRX-loaded NE.

The morphological analyses of LRX-NE as well as C-LRX NE formulations are shown in Figure 2A,B. The images obtained through TEM exhibited tiny, monodispersed, and spherical particles. Through this analysis, there is confirmation of the data from the dynamic light scattering technique, in which the introduction of chitosan appreciably magnified NE size but the overall morphology was not altered by the incorporation of chitosan.

#### 3.4.3. Globule Size Measurement

**Figure 3:**
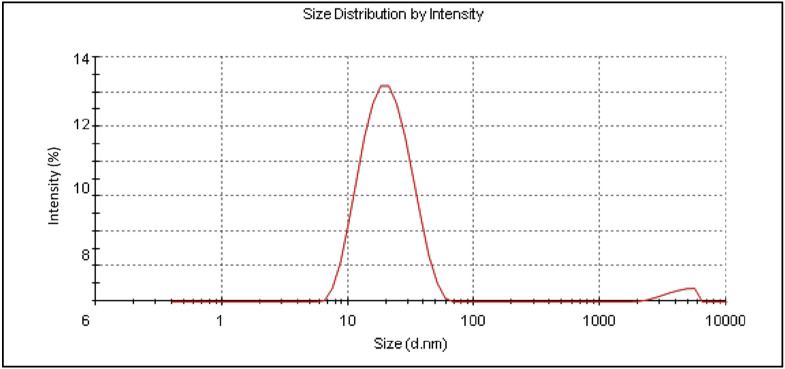
Size distribution of optimized LRX-loaded NE formulation.

Globule size for the selected NE formulations as well as the C-LRX NE formulation ranged from 63.3 ± 15.6 nm to 168.4 ± 43.2 nm (Table 4), (Figure 3), providing an acceptable range for topical NE formulations [28,29]. Polydispersity index (PDI) was observed between 0.24 ± 0.02 to 0.45 ± 0.03, exhibiting an acceptable range for such formulations [30].

#### 3.4.4. Zeta Potential Analysis

Zeta potential measurement using Zetasizer (Zetasizer Pro, Malvern, UK) generated interesting results. Zeta potential values ranged from −21.18 ± 0.15 mV to −29.58 ± 0.61 mV (Table 4). In case of the C-LRX NE formulation, a positive value of zeta potential (+20.29 ± 2010 mV) was obtained, confirming the decoration of NE with chitosan, which imparts positive charge due to its cationic nature. Generally, higher zeta potential values indicate higher stability of NEs [31]. In another study, the most stable NE formulation (NE8) showed the highest value for zeta potential (−36 mV) and exhibited good stability [16]. However, other investigations have also reported a relatively lower zeta potential (−17.95 mV), yet with stable formulation and good transdermal drug delivery [32]. In our characterization, the LRX-6 formulation has depicted the highest zeta potential value (−29.58 ± 0.61 mV).

#### 3.4.5. Viscosity and Surface Tension Measurement

Table 4 depicts the measured viscosity values for selected LRX formulations. Viscosity values range from 24.38 ± 3.97 mPa.s to 85.21 ± 2.14 mPa.s. Viscosity estimation is also a determinant of spreadability of formulation on the skin, which is also required for effective skin permeation after application [23]. Stable NEs have also reported viscosity values in a similar range [15,32], which clearly indicates that developed NE formulations, especially LRX-6, have adequate viscosity required for stability, spreadability, and transdermal absorption.

The surface tension of LRX-NE was determined by stalagmometer by counting the number of drops which moved from the starting point to the end point. The surface tension ranged from 26.46 ± 2.29 dynescm^−1^ to 56.34 ± 3.04 dynescm^−1^, as shown in Table 4. Among our selected formulations, LRX-5 and 7 exhibited values closer to human skin’s surface tension value. Our selected formulation, LRX-6, displayed a value lesser than human skin’s surface tension and could be considered a good candidate for transdermal formulation.

#### 3.4.6. pH Measurement

pH values for selected LRX-NE formulations as well as the C-LRX NE formulation were measured using a pH meter. pH values ranged from 4.73 ± 0.29 to 5.95 ± 0.35 (Table 4). Higher values (5.67 and 5.95) were estimated for LRX-6 and C-LRX NE, which are already our formulations of choice owing to suitable globule size, viscosity, and zeta potential values. These pH values correlate well with established NE formulations [28,29].

**Figure 4:**
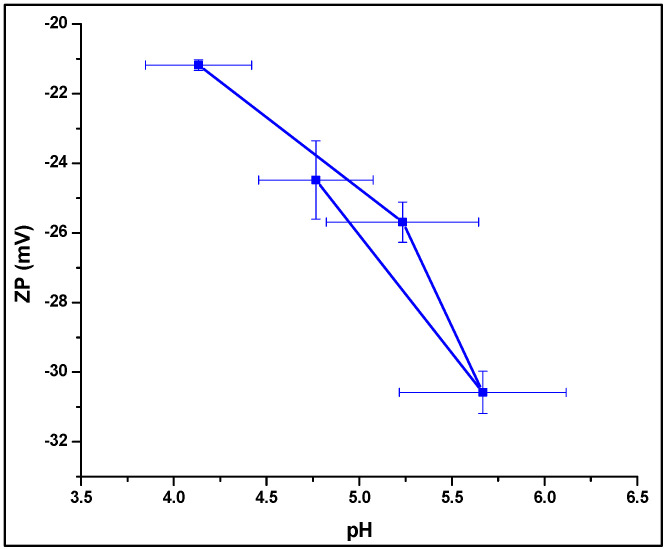
Correlation between LRX-NE formulation pH and zeta potential.

An interesting comparison could be between pH of NE formulations and zeta potential values (Figure 4). It is evident from the given data that pH increases with a decrease in zeta potential. A logical application of this correlation is difficult to establish, but decline in zeta potential and pH values corresponding to skin pH (5–6), both explain high transdermal absorption [16]. Literature also supports that formulations with pH value near to skin’s pH (5–6) display high skin permeation [33]. Of our selected formulations, LRX-5 and 6 show pH in this range (Figure 4), which is ideal for improved skin permeation [31].

#### 3.4.7. Drug Entrapment Efficiency

**Figure 5:**
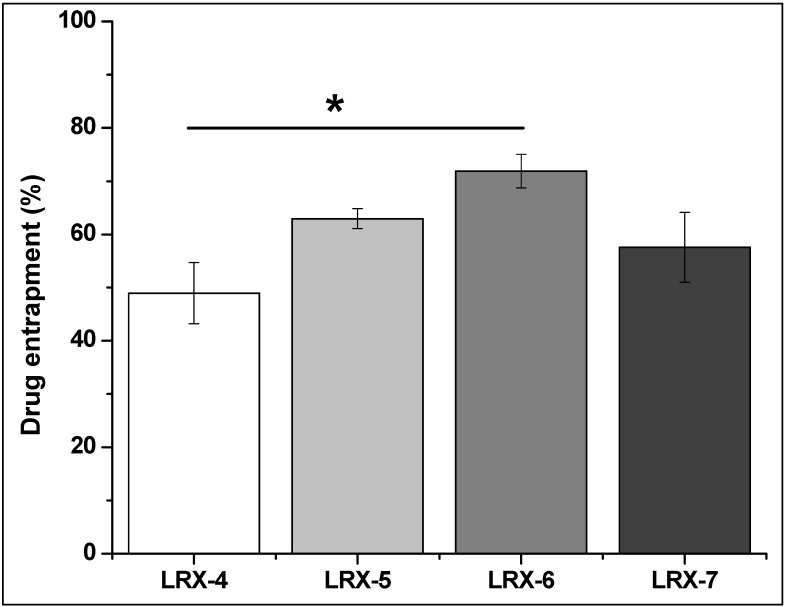
Drug entrapment efficiency of selected LRX formulations (one-way ANOVA, *p* < 0.05) LRX-6 vs. LRX-4.

The drug entrapment efficiency of selected LRX-NE formulations was evaluated using UV spectrophotometer (UV-2600i, Shimadzu, Japan) at 376 nm [14]. Good drug entrapment efficiency was found for all formulations that range between 48.96 ± 5.71% and 71.91 ± 3.17% (Table 4). LRX-6 drug entrapment efficiency was significantly different (*) than the LRX-4 formulation (*p* < 0.05) (Figure 5); no other significant difference was found between the formulations. Literature also supports such drug entrapment values [31,34]. Similarly, an optimum entrapment of LRX (65.25 ± 4.89%) was exhibited by the C-LRX NE formulation in comparison to the optimized LRX-6 NE formulation.

#### 3.4.8. In Vitro Drug Release

In vitro drug release from selected LRX formulations was evaluated using Franz Diffusion Technique [35]. Samples were taken at predetermined time intervals and the drug was analyzed at 376 nm spectrophotometrically (UV-2600i, Shimadzu, Japan). More than 50% drug release was observed for different selected formulations. The percent cumulative drug release pattern was observed in the order of LRX-6 > LRX-5 > LRX-4 > LRX-7. These values coincide with the literature’s data [32], where LRX proniosomal gel showed about 69% drug diffusion in 24 h.

**Figure 6:**
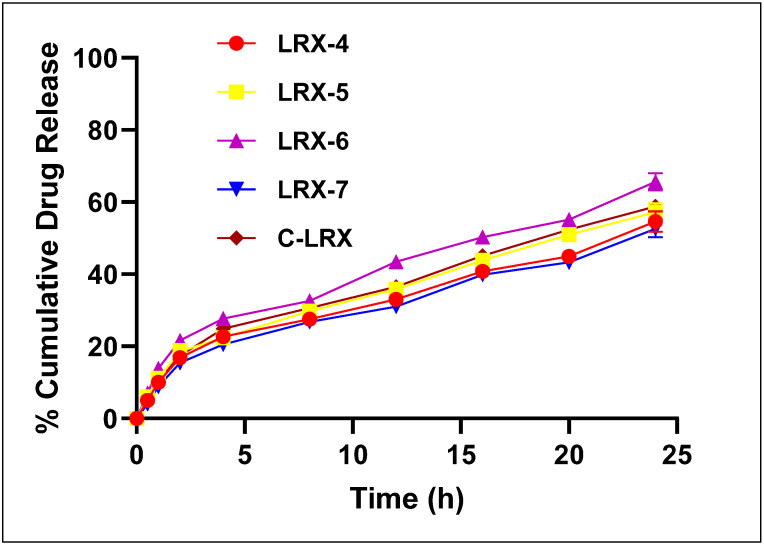
In vitro percent cumulative drug release from selected LRX-NE formulations.

Figure 6 clearly shows the trend in the release of LRX from different formulations. In vitro drug release from LRX-6 was significantly different (*p* < 0.05) from other formulations (LRX-4, 5, and 7) as evaluated by one-way ANOVA. The in vitro release profile of the optimized NE formulation of LRX (LRX-6), when compared to the NE formulation decorated with chitosan, had a slow release of LRX from the C-LRX NE formulation due to the presence of a barrier layer induced by chitosan. About 58.82% of the drug was released from the C-LRX NE formulation, producing a statistically insignificant difference from LRX-6 (*p* > 0.05).

The drug release pattern of differently formulated NEs was applied in a power law kinetic model to understand the mechanism of drug release (Table 5). “n” indicates the exponents of fraction drug release. When n’s value is greater than 0.5 and less than 1 (0.5 < n < 1), it indicates anomalous non-Fickian diffusion [36]. LRX-6 to C-LRX were less than 1 and more than 0.5 (0.5 < n < 1), which depicts a non-Fickian anomalous diffusion mechanism of drug release. Where the n value of LRX-6 is 0.597, order kinetics approach the nearly ideal zero. These results were in exact accordance with the findings of [37], which concluded that nifedipine release followed anomalous, non-Fickian diffusion.

#### 3.4.9. Ex Vivo Permeation

Different permeability parameters including permeability coefficient (Kp), steady-state flux (Jss), and enhancement ratios significantly improved in selected LRX formulations (Table 6). Our selected LRX formulation (LRX-6) exhibited the highest values for these permeability parameters, i.e., flux value of 210.16 ± 7.52 (µg cm^−2^ hr^−1^), permeability coefficient of 2.26 ± 0.077 (cm hr^−1^) ×10^−2^, and enhancement ratio of 6.85. These values were much higher compared to other selected formulations (LRX-4, 5, and 7). The literature also suggests similar higher values for permeability parameters for LRX-based transdermal preparations having high skin permeability properties [16].

**Figure 7:**
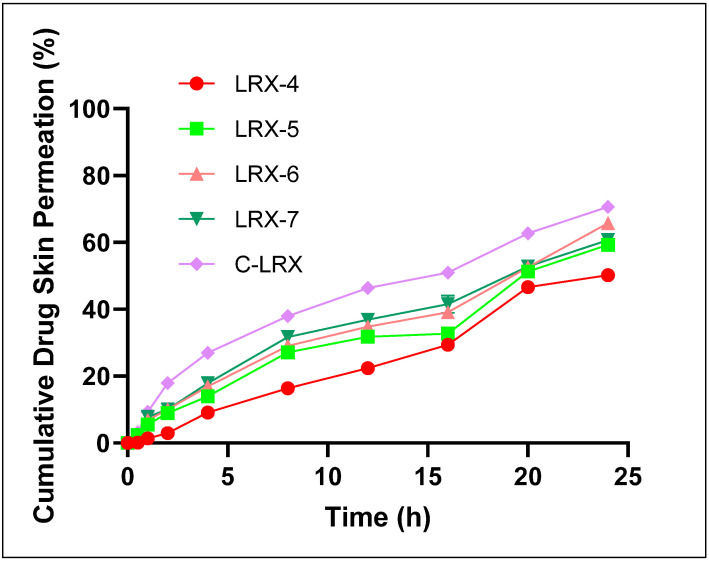
Percent cumulative drug skin permeation of selected LRX formulations.

A skin permeation study was performed on rat abdominal skin using a Franz Diffusion cell. UV spectroscopic analysis revealed appreciable skin permeation percentages for the selected formulations of LRX (Table 6). The highest cumulative skin permeation was found for LRX-6 at 65.61 ± 1.89% as compared to other selected LRX formulations (LRX-4, 5, and 7). These values and trends are consistent with the in vitro drug release profiles of these formulations (Figure 7). The good skin permeation effect of these formulations, in general, can be attributed to the skin-permeation-enhancing effects of almond oil as the oil phase [26] and Tween 80 [24,25] utilized as surfactant in the formulation composition.

Figure 7 depicts the trend in cumulative drug skin permeation through rat abdominal skin. LRX-5, 6, and 7 showed an initial incline in rate of drug skin permeation in initial approximately 7 h. This rate becomes slower later, possibly due to a decline in diffusion gradient, and later achieves the highest values in the 24 h measured samples. LRX-4 not only showed comparatively slower skin permeation in the initial phase, but also achieved the highest cumulative skin permeation value of 50.06 ± 1.31%. These values are significantly different compared to our “ideal” formulation LRX-6 (*p* < 0.05). The reason could be imbalance in quantities of almond oil and Tween 80 as compared to other formulations. The comparison in skin permeation potential and permeation parameters between optimized LRX-NE formulation (LRX-6) and C-LRX NE showed enhanced skin permeation of LRX from chitosan-decorated NE formulation, producing a statistically significant difference in percent cumulative drug permeated within 24 h as depicted in Figure 7. A similar trend was found in the case of permeation parameters as shown in Table 6. C-LRX-improved skin permeation characteristics are primarily endowed by chitosan presence, which is a crucial parameter in the skin permeation of the drugs.

## 4. Discussion

The current study aimed to develop an NE with appreciable transdermal permeation potential; therefore, an important point is to study the solubility of LRX in different oils. The solubility of LRX in oil phase can be correlated with the ability of an NE to keep the drug in dissolved form, which is also important for transdermal potential. Extensive literature survey facilitated choosing the correct oil phase for preparing the formulation intended for transdermal applications. Additionally, skin permeation is a rate-limiting step during drug absorption, where stratum corneum plays the most important impeding role. Many physical and chemical methods have been employed to improve the drug skin permeation. Alternatively, chemicals could be added that improve the skin permeation ability of formulations. Selection of a suitable type of surfactant is challenging and requires vigorous evaluation. In this regard, 300 mg of surfactant and oil phases were mixed together by heating at 50 °C for 30 min. 50 mg of this mixture was mixed with 50 mL of deionized water to prepare the emulsion. The resulting emulsion was allowed to stay for 2 h and then transmittance values were measured by UV spectrophotometry [16]. The effect of co-surfactant on the emulsification process was evaluated in a similar manner to surfactant molecules. PEG 400 not only hinders the development of rigid structures like precipitates, gels, or liquid crystals, but also improves the emulsion area [38].

A spontaneous emulsification technique/method was used for the synthesis of NEs [15,16]. In general, a hydrophobic active pharmaceutical ingredient (API) is dissolved in the oil phase and blended with the aqueous phase [17]. The mixture of both phases is then blended at high speed using a homogenizer for a short time to form a coarse oil-in-water (o/w) emulsion. Surfactant (emulsifier) molecules can be added in either the aqueous or oil phase depending on the affinity of surfactant for each phase. In case of o/w emulsion, addition of surfactant in the oil phase may impart an additional benefit of improving solubility of lipophilic substances in the oil phase. Coarse o/w emulsion is then mixed at higher speed using a high-speed homogenizer to produce fine emulsion.

Centrifugation causes creaming or sedimentation. O/w emulsions are preferred because oil exhibits reduced thickness profile compared to the aqueous phase. NE stability against gravitational force is clearly depicted by centrifugation data, which is an indirect approach. The developed C-LRX NE formulation passed the centrifugation test, clearly showing the homogeneity and stability of the system. Tween 80 (surfactant and emulsifying agent) plays a pivotal role in preventing the movement of oil globules because of centrifugal forces. As a result, the phenomena of creaming, phase separation, and aggregation are all hampered [16].

Literature suggests that the smaller the globule size, the easier it would be for an NE to penetrate through the skin. For formulations with higher globule size (>100 nm), skin permeation will depend on other physicochemical parameters, including zeta potential value, lipophilicity of drug entity, skin charges, and nature of emulsifiers [39]. Formulations with higher globule size (150 nm) are generally less likely to cross the skin barrier. However, transdermal formulations with similar globule size (>150 nm) and improved lipid character and zeta potential have also been reported [40].

It is noteworthy to mention that the presence of non-ionic surfactants (Tween 80) also imparts negative charges to the formulation. This could be due to the presence of positive (H_3_O^+^) and negative (OH^−^) charges as impurities in the system. These charges contribute to negative charges if the formulation is acidic (pH 3–6) or positive if pH is close to 9 [39].

Viscosity measurements showed a clear trend that correlates well with the parts of oil being employed in the development of the NE. It is important to mention here that the viscosity of NEs depends upon the relative concentrations of the oil phase and aqueous phase and the quantity of surfactant and co-surfactant molecules. A decrease in oil phase will relatively increase the aqueous phase and a resultant decrease in viscosity will be observed. On the other hand, a formulation with decreased quantities of surfactant and co-surfactant molecules will have a large interfacial tension in between two immiscible phases and an incline in viscosity is most likely to be observed [41,42].

It is known that skin permeation is highly dependent on the partitioning of the drug entity in-between formulation and the surface of the skin. In this regard, wettability and skin adhesion are the two important factors for achieving a milestone therapeutic outcome. Surface tension is a quantitative parameter that can help to identify the formulation’s adhesion. Human skin generally has surface tension values in the range of 27–28 dynescm^−1^. Therefore, formulations with similar or lower surface tension values are considered good candidates for showing better transdermal movement or high skin absorption.

pH values for selected LRX-NE formulations were measured using a pH meter. The highest value estimated was for LRX-6 (5.67). The pH values correlate well with established NE formulations [28,29].

The quantity of surfactant plays an important role in drug entrapment efficiency. From LRX-4 to LRX-7, as quantity of surfactant increases, drug entrapment also increases. In LRX-7, decline in drug entrapment efficiency could be due to a very low concentration of oil phase that could not hold a good quantity of drug inside and exhibited a low value (still higher than the LRX-4 formulation).

All formulations released the drug at a higher rate and around 20% of the drug was released in around 2.5 h. After, the release follows a slow and continuous pattern until all formulations show the highest percentage drug release. This release pattern is also evident in other formulations [13,15].

The good skin permeation effect of all formulations, in general, can be attributed to the skin-permeation-enhancing effects of almond oil as the oil phase [26] and Tween 80 [24,25] utilized as surfactant in the formulation’s composition.

Generally, C-LRX exhibited most parameters very close to those of the LRX-6 formulation specifically and other formulations generally, showing a stable and optimal formulation. However, C-LRX has shown improved skin permeation characteristics. It clearly showed the important role of chitosan in the skin permeation of the drugs. It is known that chitosan not only affects other parameters, but also imparts nanoemulsion surface charge. On the other hand, the presence of chitosan also allowed skin permeation via the paracellular absorption movement mechanism [37].

## 5. Conclusions

This study concludes that the poorly soluble drug lornoxicam has the potential to be used transdermally by the incorporation of lornoxicam-loaded nanoemulsion into chitosan-based nanoemulsion gel and to be further characterized by various in vitro tests. The optimized formulation of lornoxicam with and without chitosan decoration exhibited droplet size in nanoscale with uniform size distribution. The formulations passed thermodynamic stability tests, having no phase separation, cracking, or color change. Chitosan-decorated NE formulation of LRX was formulated in comparison to optimized LRX-NE formulation (LRX-6) exhibiting controlled release behavior. The use of almond oil (oil phase and penetration enhancer) and PEG 400 (co-solvent) augmented the flux of LRX through the skin. The developed C-LRX NE formulation could be expected to produce suitable analgesic and anti-inflammatory effects with no skin irritation potential. This transdermal formulation could be a suitable alternative to oral administration of LRX by producing optimum LRX therapeutic levels with enhanced patient compliance as well as reduced dosing frequency and gastrointestinal-related adverse effects.

## Figures and Tables

**Table 1:** Lornoxicam solubility in selected oils, surfactants, and co-surfactants.

Ingredients		Solubility (mg mL^−1^) Mean ± SD
Oils	Almond oil	0.035 ± 0.004
	Coconut oil	0.029 ± 0.003
	Olive oil	0.011 ± 0.007
	Sesame oil	0.0312 ± 0.002
	Sunflower oil	0.048 ± 0.006
Surfactants	Cremophor RH 40	5.05 ± 0.056
	Tween 80	3.33 ± 0.037
Co-surfactants	DMSO	7.00 ± 0.067
	Ethanol	0.085 ± 0.018
	PBS (pH 7.4)	6.1 ± 0.021
	PEG 400	4.132 ± 0.02
	Water	0.025 ± 0.008

Data are expressed as mean ± SD, n = 3.

**Table 2:** Formulations of LRX prepared with varying ratios of oil and surfactant molecules.

Formulation Code	Oil	Surfactant (S)	Co-Surfactant (Co-S)	S: Co-S Ratio	Parts of Oil	Parts of Surfactant	(Oil: S Mix) %
LRX-1	Almond oil	Tween 80	Ethanol	2:1	9	1	9.0
LRX-2					8	2	4.0
LRX-3					7	3	2.3
LRX-4					6	4	1.5
LRX-5					5	5	1.0
LRX-6					4	6	0.7
LRX-7					3	7	0.4
LRX-8					2	8	0.3
LRX-9					1	9	0.1
C-LRX (2%)					4	6	0.7

**Table 3:** Physical characterization of optimized Blank, LRX-NE (LRX-6), and C-LRX NE formulations at various temperatures.

F. Codes	Temperature	Color	Odor Change	Phase Separation	Centrifugation Stability	Thermodynamic Test
Blank	4 °C	White	No change	Nil	Stable	Passed
	25 °C	White	No change	Nil	Stable	Passed
	45 °C	White	No change	Nil	Stable	Passed
Optimized LRX-NE	4 °C	Pale Yellow	No change	Nil	Stable	Passed
	25 °C	Pale Yellow	No change	Nil	Stable	Passed
	45 °C	Pale Yellow	No change	Nil	Stable	Passed
C-LRXNE	4 °C	Yellow	No change	Nil	Stable	Passed
	25 °C	Yellow	No change	Nil	Stable	Passed
	45 °C	Yellow	No change	Nil	Stable	Passed

Data are expressed as mean ± SD, n = 3.

**Table 4:** Physicochemical characterization of selected LRX formulations.

Formulation Code	Globule Size (nm ± SD)	PDI	ZP (mV ± SD)	Viscosity (mPa.s ± SD)	Surface Tension (Dynescm^−1^)	pH Mean ± SD	Drug Entrapment Efficiency (%) Mean ± SD
LRX-4	168.4 ± 43.2	0.45 ± 0.03	−21.18 ± 0.15	85.21 ± 2.14	56.34 ± 3.04	4.73 ± 0.29	48.96 ± 5.71
LRX-5	125.8 ± 36.5	0.25 ± 0.04	−25.69 ± 0.58	62.19 ± 3.18	39.59 ± 4.08	5.23 ± 0.41	62.95 ± 1.90
LRX-6	78.6 ± 11.7	0.24 ± 0.02	−29.58 ± 0.61	48.47 ± 2.12	26.46 ± 2.29	5.67 ± 0.45	71.91 ± 3.17
LRX-7	63.3 ± 15.6	0.31 ± 0.06	−24.48 ± 1.12	24.38 ± 3.97	35.01 ± 4.77	4.47 ± 0.31	57.58 ± 6.58
C-LRX	101.3 ± 24.51	0.27 ± 0.04	+20.29 ± 2.10	58.12 ± 5.09	31.05 ± 3.57	5.95 ± 0.35	65.25 ± 4.89

Data are expressed as mean ± SD, n = 3.

**Table 5:** Kinetic modeling of selected LRX-NE formulations.

Formulations	Power Law Kinetic Model			
	K ± SD	R^2^	N	Release Mechanism
LRX-4	0.263 ± 0.131	0.8176	0.432	Fickian Diffusion
LRX-5	0.032 ± 0.024	0.9657	0.501	Anomalous non-Fickian Diffusion
LRX-6	0.179 ± 0.015	0.9751	0.597	Anomalous non-Fickian Diffusion
LRX-7	0.021 ± 0.001	0.9122	0.405	Fickian Diffusion
C-LRX NE	0.089 ± 0.156	0.9976	0.583	Anomalous non-Fickian Diffusion

**Table 6:** Permeability parameters for selected LRX-NE formulations.

Formulation Code	Steady-State Flux Jss ± S (µg cm^−2^ hr^−1^)	Permeability Coefficient Kp ± SD (cm hr^−1^) × 10^−2^	Enhancement Ratio (ER)
LRX-4	45.63 ± 3.15	0.35 ± 0.019	1.63
LRX-5	95.63 ± 5.67	2.36 ± 0.15	3.21
LRX-6	210.16 ± 7.52	2.26 ± 0.077	6.85
LRX-7	121.25 ± 3.65	1.12 ± 0.015	4.22
C-LRX NE	229.18 ± 9.25	2.49 ± 0.127	7.64

Data are expressed as mean ± SD, n = 3.

## Data Availability

Not applicable.
